# Sustained Impact of RHDV2 on Wild Rabbit Populations across Australia Eight Years after Its Initial Detection

**DOI:** 10.3390/v15051159

**Published:** 2023-05-12

**Authors:** David S. Ramsey, Kandarp K. Patel, Susan Campbell, Robyn N. Hall, Patrick L. Taggart, Tanja Strive

**Affiliations:** 1 Arthur Rylah Institute, Department of Energy, Environment and Climate Action, Heidelberg, VIC 3083, Australia; david.ramsey@delwp.vic.gov.au; 2 Biosecurity, Department of Primary Industries and Regions (PIRSA), Urrbrae, SA 5064, Australia; kandarp.patel@adelaide.edu.au; 3 Centre for Invasive Species Solutions, The University of Canberra, Bruce, ACT 2617, Australia; robyn.hall@csiro.au (R.N.H.); patrick.taggart@bushheritage.org.au (P.L.T.); 4 School of Animal and Veterinary Sciences, The University of Adelaide, Roseworthy, SA 5371, Australia; 5 Invasive Species and Environment Biosecurity, Department of Primary Industries and Regional Development, Albany, WA 6330, Australia; susan.campbell@dpird.wa.gov.au; 6 Commonwealth Scientific and Industrial Research Organisation, Health and Biosecurity, Canberra, ACT 2601, Australia; 7 Vertebrate Pest Research Unit, Department of Primary Industries NSW, Queanbeyan, NSW 2800, Australia

**Keywords:** Lagovirus, viral competition, wildlife diseases, biocontrol

## Abstract

Following the arrival of rabbit haemorrhagic disease virus 2 (RHDV2) in Australia, average rabbit population abundances were reduced by 60% between 2014 and 2018 based on monitoring data acquired from 18 sites across Australia. During this period, as the seropositivity to RHDV2 increased, concurrent decreases were observed in the seroprevalence of both the previously circulating RHDV1 and RCVA, a benign endemic rabbit calicivirus. However, the detection of substantial RHDV1 seropositivity in juvenile rabbits suggested that infections were continuing to occur, ruling out the rapid extinction of this variant. Here we investigate whether the co-circulation of two pathogenic RHDV variants was sustained after 2018 and whether the initially observed impact on rabbit abundance was still maintained. We monitored rabbit abundance and seropositivity to RHDV2, RHDV1 and RCVA at six of the initial eighteen sites until the summer of 2022. We observed sustained suppression of rabbit abundance at five of the six sites, with the average population reduction across all six sites being 64%. Across all sites, average RHDV2 seroprevalence remained high, reaching 60–70% in adult rabbits and 30–40% in juvenile rabbits. In contrast, average RHDV1 seroprevalence declined to <3% in adult rabbits and 5–6% in juvenile rabbits. Although seropositivity continued to be detected in a low number of juvenile rabbits, it is unlikely that RHDV1 strains now play a major role in the regulation of rabbit abundance. In contrast, RCVA seropositivity appears to be reaching an equilibrium with that of RHDV2, with RCVA seroprevalence in the preceding quarter having a strong negative effect on RHDV2 seroprevalence and vice versa, suggesting ongoing co-circulation of these variants. These findings highlight the complex interactions between different calicivirus variants in free-living rabbit populations and demonstrate the changes in interactions over the course of the RHDV2 epizootic as it has moved towards endemicity. While it is encouraging from an Australian perspective to see sustained suppression of rabbit populations in the eight years following the arrival of RHDV2, it is likely that rabbit populations will eventually recover, as has been observed with previous rabbit pathogens.

## 1. Introduction

Rabbit haemorrhagic disease virus 2 (RHDV2) emerged as a novel rabbit calicivirus in 2010 in Europe [1,2]. Following its emergence, it rapidly spread across the globe and is now affecting domestic and wild lagomorph populations worldwide [3,4,5,6].

Similar to the previously known RHDV of rabbits (termed RHDV1), recent isolates of RHDV2 are highly virulent, causing infectious hepatitis with high case fatality rates in susceptible rabbits (*Oryctolagus cuniculus*) [7]. However, in contrast to RHDV1, RHDV2 has a broader host range within the order Lagomorpha and is able to infect various species of hares (*Lepus* sp.) and cottontails (*Sylvilagus* sp.) [8,9,10], and can fatally infect rabbits that are resistant to infection with virulent RHDV1 at a very young age [11,12]. Furthermore, recent work has shown that this ability to infect young rabbits enables RHDV2 to amplify in susceptible populations earlier [13]. Together with the ability to overcome pre-existing immunity to RHDV1, these attributes have been proposed as a key factor driving RHDV2′s epidemiological competitiveness over RHDV1, resulting in its global spread.

The impact of RHDV2 has been substantial. For domestic and farmed rabbits, strict biosecurity measures were the only effective protection against RHDV2 until specific vaccines were produced [14]. For lagomorph populations in their native range, RHDV2 is of conservation concern. For example, rabbits are a key food resource for specialised native predators in the Iberian Peninsula [15], and in the Americas, many native lagomorph species are susceptible to RHDV2 [6,10]. In contrast, highly virulent, self-disseminating and species-specific rabbit viruses have been deliberately introduced several times during the last seven decades in Australia to mitigate the substantial economic and environmental impact of introduced European rabbits [16,17,18].

In Australia, RHDV2 was first reported in May 2015 [19], but retrospective serological testing and genetic analysis estimated its likely arrival on the Australian continent up to 6 to 18 months earlier [20,21]. The incursion of RHDV2 coincided with preparations for the nationwide release of an additional strain of RHDV1 (RHDV-K5), a naturally occurring RHDV variant from Korea. The aim of this release was to ‘boost’ the waning effectiveness of naturally circulating RHDV1, which had been released in the mid-1990s [22]. As part of this planned rollout, a national rabbit monitoring network was operating at the time of RHDV2’s arrival in Australia. This network provided an excellent opportunity to track RHDV2′s spread and impact, as well as the interaction and competition of several co-circulating viruses in rabbits. An opportunistic sampling of dead rabbits and molecular epidemiology studies documented the swift spread of RHDV2 across the continent, where it became the dominant virus within 18 months [19]. Parallel surveys at the long-term rabbit monitoring sites, where population abundance was measured and up to 20 apparently healthy rabbits were shot and sampled several times annually for serological analysis, facilitated in-depth studies of RHDV2 impacts as it spread across the continent from east to west [23,24].

Analyses of the data from the national rabbit monitoring network until 2018 revealed that the arrival and subsequent spread of RHDV2 resulted in an average reduction in the abundance of wild rabbits by 60% [23], with impacts most pronounced in South Australia [25] and Western Australia. In addition, these studies revealed that the seroprevalence of RHDV2 rapidly increased following its emergence, with concurrent decreases in the seroprevalence of both RHDV1 and RCVA, a benign endemic rabbit calicivirus. However, the serological analysis did not support the rapid extinction of the RHDV1 variant; substantial increases in juvenile RHDV1 seroprevalence indicated that infections were continuing to occur.

Following the conclusion of the study by Ramsey et al. (2020) [23], rabbit spotlight monitoring and serological sampling continued post-autumn 2018 at a reduced subset of the original monitoring sites to determine whether the suppression of rabbit populations following the arrival of RHDV2 continued once the virus became endemic. In addition, estimation of the trends in seroprevalence of RHDV2, RHDV1 and RCVA from these sites should reveal whether the declining trends in seroprevalence of RHDV1 and RCVA have continued. In particular, we hypothesised that RHDV1 seroprevalence would continue to decline or indeed disappear from the landscape as has occurred elsewhere [2,26]. However, whether RCVA continued to be negatively impacted by competition with RHDV2 remained an open question. Hence, analysis of the comprehensive monitoring data from these sites should enable further insights into the interactions between the three endemic viruses, all of which compete for the same host.

## 2. Methods

### 2.1. Spotlight Monitoring

**Figure 1:**
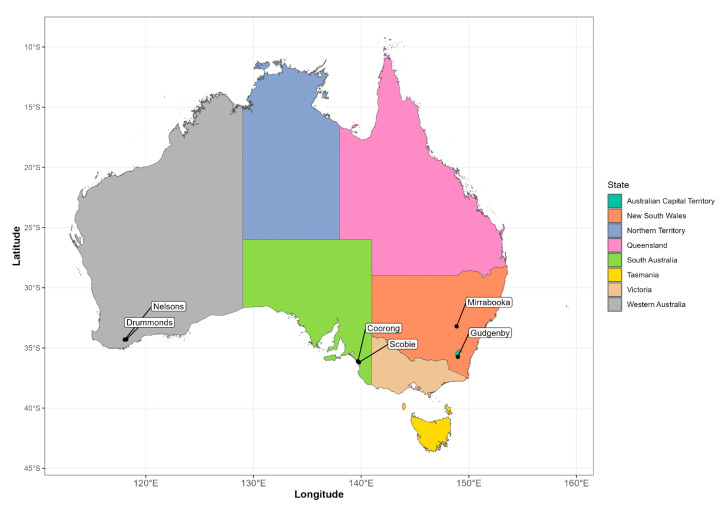
Locations of the six rabbit monitoring sites where rabbit spotlight counts and serum sampling were conducted between 2018–2022.

Monitoring of rabbit abundance post-2018 was undertaken at six of the original 18 national monitoring network sites, Mirrabooka (NSW), Gudgenby (ACT), Coorong and Scobie (SA), and Nelsons and Drummonds (WA) (Figure 1). Monitoring consisted of spotlight counts conducted quarterly, in approximately February (summer), April (autumn), July (winter) and October (spring). Spotlight counts occurred along transects using a hand-held 150 W light from the rear of a utility vehicle driven at slow speed during the early evening. Transect lengths at each site varied from 1.0 to 20.0 km. The number of rabbits seen on the transect was recorded each night for up to three consecutive nights. Spotlight count data were collected up to and including summer 2022 but were incomplete to various degrees for each of the six sites due to unforeseen events (bushfire, flooding, other adverse weather). A summary of the post-autumn 2018 monitoring record for the six sites is given in Table S1 (Supplementary Materials). In addition to the collection of the post-2018 rabbit spotlight data, we also accessed considerable historical spotlight data (between 2006 and 2015) from the Gudgenby site; these data were not originally available in the analysis of Ramsey et al. (2020) [23]. However, for the current analyses, only data from 2011 were considered so that the time series was consistent with other comparable sites.

### 2.2. Serology

Following the completion of spotlight counts at each site, serum samples were collected from up to 20 shot rabbits from areas in the vicinity of each transect. Serum samples collected post-2018 were undertaken at five of the same six monitoring sites; no serum samples were obtained from the Coorong site in SA. Serum samples were screened by a series of enzyme-linked immunosorbent assays (ELISAs) to determine the presence of RHDV1 or RHDV2 antibodies as well as antibodies to RCVA, as described previously [20,23]. Due to various levels of cross-reactivity between the respective competition ELISAs, rabbits were scored as positive to RHDV1 (including classical RHDV and RHDV-K5) if the ratio of the RHDV2/RHDV1 cELISA reciprocal titres was <1. Similarly, rabbits were scored positive for RHDV2 if the ratio was >1. For RHDV1 and RHDV2 cELISAs, titres ≥1:40 were considered to be positive, while a titre of 1:20 on the blocking ELISA was considered to be positive for RCVA. The age of each rabbit was also estimated using dried eye lens weight, which is accurate up to approximately 500 days of age [27]. Rabbits were then classified as juveniles (≤150 days) or adults (>150 days).

### 2.3. Analysis of Rabbit Spotlight Data

An equivalent dynamic state-space model to that detailed in Ramsey et al. (2020) [23] was fitted to the spotlight count data that included the post-2018 data from all six sites and the additional historical data for the Gudgenby site. Briefly, the rabbit counts at each site i at occasion t and night k ($y_{itk}$) were corrected for imperfect detectability to estimate absolute rabbit abundance at site i and time t ($N_{it}$) using a dynamic N-mixture model [28].

$$y_{itk}\;\sim \;Bin\left(p_{it},\;N_{it}\right)$$

(1) $$N_{it}\;\sim \;Poisson\left(\mu_{it}\cdot T_{it}\right),$$

where $p_{it}$ is the detection probability of rabbits at site i and occasion t and $\mu_{it}$ is the expected population abundance of rabbits. The length of each transect monitored at each site and occasion ($T_{it}$) was included as an offset in the N-mixture model to account for the variable length of transects (km) monitored at each site. The time series of rabbit abundance estimates $\mu_{it}$ were then used to predict the dynamics of the rabbit populations at each site using a hierarchical, Bayesian state-space modelling approach. The population model consisted of a discrete-time, stochastic Gompertz model of density-dependent population regulation [29].

(2) $$\begin{array}{ll} \ln(\mu_{it}) & =\ln(\mu_{it-1})+r_{it}\\ r_{it} & =a_{i}+b_{i}\cdot \ln\left(\mu_{it-1}\right)+\delta S_{it}+\gamma_{i}R_{it}+\eta_{i}K_{it}+e_{t} \end{array},$$

where $r_{it}$ is the rate of increase for site i between time t−1 and time t, $a_{i}$ is the intrinsic (maximum) rate of increase, $b_{i}$ governs the strength of density-dependence and $e_{t}$ is the stochastic process error at time t. In addition to these demographic parameters, we also investigated the effect of the season (S), the arrival of RHDV2 (R) and the release of RHDV-K5 (K) on rabbit rate of increase where $\delta ,\;\gamma_{i}$ and $\eta_{i}$ are parameters to be estimated.

In particular, we wished to estimate equilibrium abundance (i.e., carrying capacity—κ) both before ($\kappa^{b}$) and after ($\kappa^{a}$) the arrival of RHDV2. These were estimated by

(3) $$\kappa_{i}^{b}=-\left(a_{i}+\delta \right)/b_{i}$$

(4) $$\kappa_{i}^{a}=-\left(a_{i}+\delta +\gamma_{i}\right)/b_{i},$$

where $\kappa_{i}^{b}$ and $\kappa_{i}^{a}$ are the equilibrium abundances for rabbits during the winter-spring months for each site i before and following the arrival of RHDV2. More details on the state-space model are provided by Ramsey et al. (2020) [23]. Estimates of population suppression (reductions in equilibrium abundance) were then examined for the six sites and compared to the results of Ramsey et al. (2020) [23].

### 2.4. Serological Analysis

Exponential growth state-space models were fitted to the time series of serological prevalence for the three variants (RCVA, RHDV1 and RHDV2) to examine trends in age-specific seroprevalence for the period following the arrival of RHDV2 [23].

(5) $$n_{akit}\;\sim \;Bin\left(p_{akit},\;N_{akit}\right)$$

$$\mathrm{logit}\left(p_{akit}\right)=x_{akit}$$

$$x_{akit}=x_{akit-1}+A_{ak}+\varepsilon_{ak}+\eta_{ik}$$

$$\varepsilon_{ak}\;\sim \;N\left(0,\;\;\sigma_{ak}\right);\;\;\eta_{ik}\;\sim \;N\left(0,\;\;\sigma_{i}\right)$$

where $n_{akit}$, $N_{akit}$ and $p_{akit}$ are the number of rabbits testing positive, the number of rabbits tested, and the antibody prevalence, respectively, for age class a and variant *k* at site i and time *t* with $A_{ak}$ representing the average trend for age class a and variant *k* (on the logit scale). The parameters $\varepsilon_{ak}$ represent the process errors for each age class and variant, while the $\eta_{ik}$ represent random effects for each site and variant. We also examined the potential interactions both within and between each variant by using a multivariate autoregressive (MAR) version of Equation (5) with a single time lag (i.e., MAR(1)), which examines the effect of a variant at time *t*−1 on a variant at time *t* [30]. Using matrix notation for the logit transformed vector of prevalence values for each variant at site *i* and time *t* ($x_{it}$), the state process was given by

(6) $$x_{it}=A_{i}+{\mathrm{Bx}}_{it-1}+u_{it-1}+w_{t}$$

where $A_{i}$ was a 3 × 1 vector of constants for each site *i*, and B was a 3 × 3 matrix of parameters whose elements $b_{kk^{\prime }}$ relate the effect of variant *k* on itself between times *t*−1 and time *t* ($k=k^{\prime }$) as well as the interaction between variant k and variant $k^{\prime }$ between times *t* and *t* − 1 ($k\neq k^{\prime }$). More details on the models fitted to the seroprevalence data are provided by Ramsey et al. (2020) [23].

### 2.5. Model Fitting

The state-space model (Equations (1)–(4)) as well as the serological models (Equations (5) and (6)) were fitted to the data using the Bayesian Markov chain Monte Carlo (MCMC) software Stan [31]. Weakly-informative half-*t*_4_ priors were specified for all standard deviation parameters, and weakly informative $N\left(0,\;5\right)$ priors were specified for all regression parameters. WIn addition; we used a weakly informative prior for the logit-transformed detection probability parameters, specified as $N\left(0,\;1.6\right)$. For the MAR(1) model, we used a prior for the correlation matrix Ω instead of the covariance matrix Σ using the LKJ prior for correlation matrices [32], setting the shape parameter to 1, which is equivalent to a uniform density for all correlations.

The convergence of the MCMC algorithms was assessed using the scale-reduction diagnostic of Brooks & Gelman [33] and by visual inspection of parameter trace plots. First, a burn-in of 2000 iterations was undertaken, followed by sampling from five independent Markov chains with different starting values for 2000 further iterations giving a total of 10,000 samples for each parameter for inference. Data and R source code used to fit the state-space and serology models are archived at Zenodo [34].

## 3. Results

### 3.1. Spotlight Data

**Figure 2:**
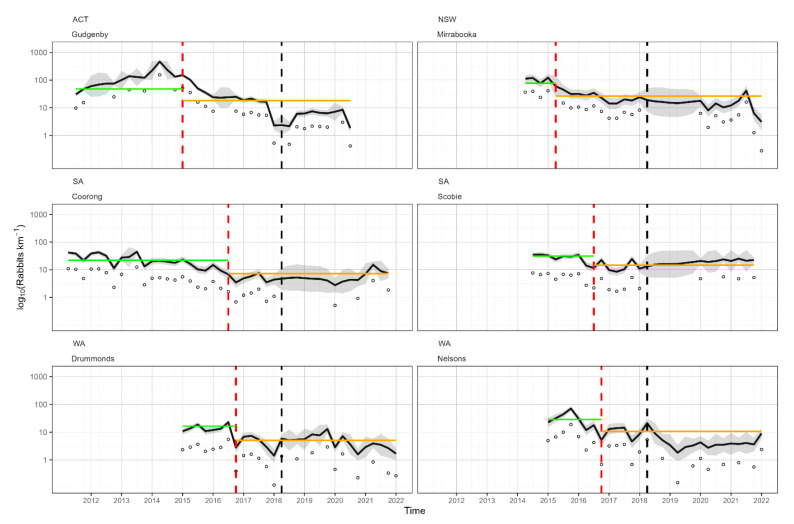
Predicted abundances (solid black lines) and observed counts (grey points) of rabbits (rabbits/spotlight km) at each of the six sites monitored between 2011 and 2022. Solid black lines are posterior medians, and shaded grey polygons are the 95% credible intervals of expected abundances. The dashed red vertical line indicates the arrival time of RHDV2 at the site based on serology (Ramsey et al., 2020) [23]. The dashed vertical black line shows the start of new data added since Ramsey et al. (2020) [23]. Horizontal green and orange lines give the estimates of $\kappa^{b}$ (equilibrium rabbit abundance before RHDV2 arrival) and $\kappa^{a}$(equilibrium rabbit abundance after RHDV2 arrival), respectively, for sites with at least one year of monitoring data prior to the arrival of RHDV2. y-axes are $\log_{10}$ transformed.

Population trajectories of rabbit abundance for the six sites ranged between one and 495 rabbits per spotlight km (Figure 2). The highest rabbit abundances were recorded at Gudgenby (ACT) prior to the arrival of RHDV2, and the lowest abundances were recorded at Drummonds (WA) post the arrival of RHDV2. Estimates of equilibrium population abundance indicated that the overall average suppression of rabbits since the arrival of RHDV2 was 64%, similar to that estimated by Ramsey et al. (2020) [23] (Table 1). Of the six sites with post-2018 monitoring data, the highest suppression of abundance was recorded at the Gudgenby site (82%) and the lowest at Scobie (52%). However, there was high uncertainty around the estimated suppression at the Scobie site, which included the possibility that no suppression of rabbit abundance occurred (Table 1).

### 3.2. Serological Data

**Figure 3:**
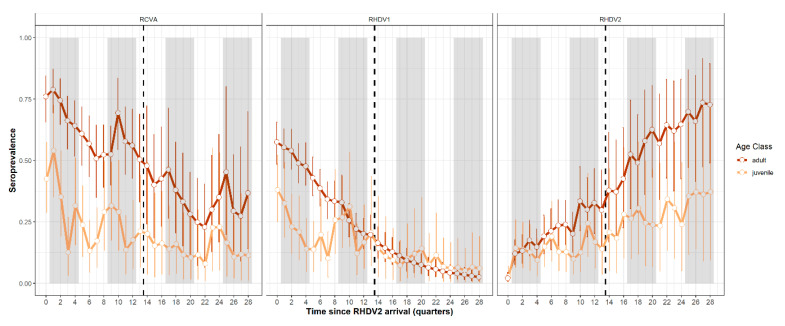
Estimated average trends in the seroprevalence of RCVA, RHDV1 and RHDV2 for juvenile (≤150 days old) and adult (>150 days) rabbits following the arrival of RHDV2 at each site. The black vertical line indicates the start of new data added since Ramsey et al. (2020) [23].

Average trends in seroprevalence from the five sites with post-2018 serum samples revealed that RHDV1 seroprevalence continued to decline, especially in adult rabbits (Figure 3). However, there was some evidence of recent RHDV1 seropositivity in juvenile rabbits (Figure 3), especially at the Scobie site in South Australia (Figure S1—Supplementary Materials). In contrast, RHDV2 seroprevalence continued to increase, with an average adult seroprevalence of approximately 60–70% post-2018 and juvenile seroprevalence of approximately 30–40% over the same time period (Figure 3 and Figure S2—Supplementary Materials). In contrast to RHDV1, the seroprevalence of RCVA, while declining initially, has shown recent signs of stabilising in both juveniles and adults (Figure 3). The exception to this is the Gudgenby site, where RCVA seroprevalence has declined over the last two years (Figure S3—Supplementary Materials).

Estimates of the interactions between variants (Equation (6)) indicate that RCVA seroprevalence in the previous quarter had a strong negative effect on RHDV2 seroprevalence, while the reciprocal effect of RHDV2 on RCVA seroprevalence was also evident (Table 2). This differs from the findings of Ramsey et al. (2020) [23], where there was a strong negative effect of RHDV2 on RCVA seroprevalence and a weak reciprocal effect. Hence, this suggests that competition between RCVA and RHDV2 is becoming more balanced, providing firmer evidence for the coexistence between these two variants. In contrast, RHDV1 continues to be at a competitive disadvantage due to the continued strong negative effects of RHDV2 on RHDV1 seroprevalence (Table 2). However, although RHDV1 seroprevalence continues to decline, the serological data does not indicate its extinction.

## 4. Discussion

Analysis of additional spotlight monitoring and serological data from rabbit populations at 6 of the original 18 long-term monitoring sites has revealed additional insights into the interactions and effects of RCVA/RHDV1/RHDV2 infections on rabbit populations. Rabbit populations at all monitored sites, with the exception of Scobie, were suppressed compared to pre-RHDV2 arrival populations, with the average population reduction across all six sites being 64%. As predicted, the serological data show that RHDV2 continues to be the dominant calicivirus variant in rabbit populations with a seroprevalence of 60–70% in adult rabbits, consistent with findings from recent molecular epidemiology and serology studies [21,35]. Although the seroprevalence of RCVA was initially depressed by competition with RHDV2 [23], RCVA seroprevalence now appears to be stabilising. A likely explanation for this is reduced competition for the infection of juvenile rabbits from RHDV2 now that it has become endemic. Recent molecular analysis of the time series of the different calicivirus variants has indicated that the peak activity of RHDV2 has probably passed [21]. Transmission of RCVA is thought to be sustained by young rabbits that become infected soon after weaning [36,37]. These new cohorts were severely affected by RHDV2 during the early phases of its spread and establishment. Now that it is establishing endemicity, high levels of population immunity to RHDV2 translate to the high prevalence of RHDV2-specific maternal immunity in young rabbits, which has been shown to prevent lethal disease (but not infection) in this age cohort [7]. This may result in sufficient numbers of young rabbits remaining alive for long enough to sustain RCVA circulation at several sites. Interestingly, the stronger support now evident for a negative effect of RCVA on RHDV2 seroprevalence in the following quarter suggests some level of cross-protection may be evident, similar to that observed between RCVA and RHDV1, which was found to be partial and transient [38]. Experimental infection studies have shown cross-protection against fatal RHDV2 infection following recent RCVA exposure, which suggests that cross-protection between heterologous rabbit caliciviruses, in general, appears transient and declines with increasing time between infections [39]. If indeed similar to the cross-protection observed for RCVA and RHDV1, it is also feasible that previous RCVA infection not just reduces case fatality rates but also infection rates [40].

The seroprevalence of RHDV1 continued to decline at monitored sites, approaching 0% seroprevalence in adult rabbits. Hence, it is unlikely that RHDV1 strains now play a major role in the regulation of rabbit abundance. Nevertheless, some residual infections in juvenile rabbits suggest that the RHDV1 variants continue to occur in some areas, especially in South Australia. However, this conclusion should be tempered by the low number of juvenile rabbits subject to serological testing, which was evidenced by the high uncertainty in prevalence estimates for RHDV1, especially in the Western Australian sites. It also needs to be noted that the RHDV-K5 virus continues to be deliberately released across the continent [13], which may have contributed to some of these observations. Further analysis is currently underway to determine if RDHV-K5 has become established in some wild rabbit populations.

While it is encouraging from an Australian perspective to see the initial suppression of wild rabbit populations by RHDV2 sustained eight years after its initial emergence, previous experiences with myxoma virus and RHDV1 biological control have shown that rabbit populations invariably start to recover [41,42]. This may be due to changes in the epidemiology of RHDV2 influencing levels of population immunity (e.g., [38]), as well as host-pathogen co-evolution leading to varying levels of heritable genetic resistance [43]. While such increased, heritable genetic resistance will be welcome in regions where RHDV2 impacts native lagomorph populations, in the Australian pest management context this means that integration of currently available conventional and biological tools as well as the search for new management tools and strategies for rabbit control must continue [22].

## Figures and Tables

**Table 1:** Estimates of equilibrium rabbit abundances (κ) during winter-spring (rabbits/km) for the period prior to (${\hat{\kappa }}^{b}$) and following (${\hat{\kappa }}^{a}$) the arrival of RHDV2, and the proportional change in the equilibrium abundance (Δ). Estimates are presented for the six sites with post-2018 monitoring data. The average estimate includes all sites with at least one year of monitoring data prior to the estimated arrival of RHDV2. Se—standard error; LCL—lower 95% credible interval; UCL—upper 95% credible interval.

Scheme.	${\hat{\kappa }}^{b}$	$\mathrm{se}\left(\kappa_{b}\right)$	${\hat{\kappa }}^{a}$	$\mathrm{se}\left(\kappa_{a}\right)$	Δ	LCL(Δ)	UCL(Δ)
Coorong	20.3	4.7	7.3	2.4	**−0.64**	−0.85	−0.34
Drummonds	15.4	5.0	5.8	1.9	**−0.61**	−0.86	−0.32
Gudgenby	137.3	59.2	23.6	11.3	**−0.82**	−0.96	−0.55
Mirrabooka	66.5	32.9	21.1	8.0	**−0.68**	−0.92	−0.24
Nelsons	27.5	10.5	8.7	3.6	**−0.67**	−0.89	−0.33
Scobie	31.2	11.0	15.6	6.0	**−0.52**	−0.80	0.17
Average	39.9	30.2	15.4	11.3	**−0.64**	−0.91	−0.01

**Table 2:** Table of parameter estimates describing the effect of antibody prevalence for a variant at time *t* − 1 (columns) on the change in antibody prevalence for a variant at time *t* (rows). Values in bold indicate interactions between variants that have 95% credible intervals that do not include zero. Diagonal entries describe the self-effects of a variant at time *t* − 1 on the same variant at time *t*.

Variant	RCVA	RHDV1	RHDV2
**RCVA**	0.285 [0.162, 0.406]	0.021 [−0.156, 0.193]	**−0.215 [−0.319, −0.119]**
**RHDV1**	**0.128 [0.023, 0.235]**	0.112 [−0.051, 0.278]	**−0.164 [−0.251, −0.082]**
**RHDV2**	**−0.166 [−0.295, −0.048]**	**0.271 [ 0.103, 0.45]**	0.749 [0.649, 0.844]

## Data Availability

Data and code ara available online: https://zenodo.org/record/3546398 (accessed on 17 April 2023).

## Supplementary Materials

The following supporting information can be downloaded at: https://www.mdpi.com/article/10.3390/v15051159/s1, Table S1: Serological sampling in the period following Autumn 2018 at five sites; Figure S1: Estimated average trends in the seroprevalence of RHDV1 for juvenile and adult rabbits; Figure S2: Estimated average trends in the seroprevalence of RHDV2 for juvenile and adult rabbits; Figure S3: Estimated average trends in the seroprevalence of RCVA for juvenile and adult rabbits.
