# Supplementary material for: Sustained Impact of RHDV2 on Wild Rabbit Populations across Australia Eight Years after Its Initial Detection
Source: Viruses. 2023 May 12;15(5):1159. doi: 10.3390/v15051159 (PMC10223972; doi:10.3390/v15051159)
Supplement: Supplementary file 1 [file viruses-15-01159-s001.zip › viruses-2377489-supplementary.pdf]

Supplementary materials

Table S1. Serological sampling in the period following Autumn 2018 at five sites. Su – summer; Au – autumn; Wi – winter; Sp – spring.

Site	Year	Seasons
**Mirrabooka (NSW)**	2020	Su, Au, Wi, Sp
	2021	Su, Au, Wi
	2022	Su
**Gudgenby (ACT)**	2006-2015	
	2018	Wi, Sp
	2019	Su, Au, Wi, Sp
	2020	Wi, Sp
	2021	Su, Au
	2022	Su
**Scobie (SA)**	2020	Au, Sp
	2021	Au, Sp
**Drummonds (WA)**	2018	Sp
	2019	Au, Sp
	2020	Au, Sp
	2021	Su, Au
	2022	Su
**Nelsons (WA)**	2018	Sp
	2019	Au, Sp
	2020	Au, Sp
	2021	Su, Au, Sp
	2022	Su

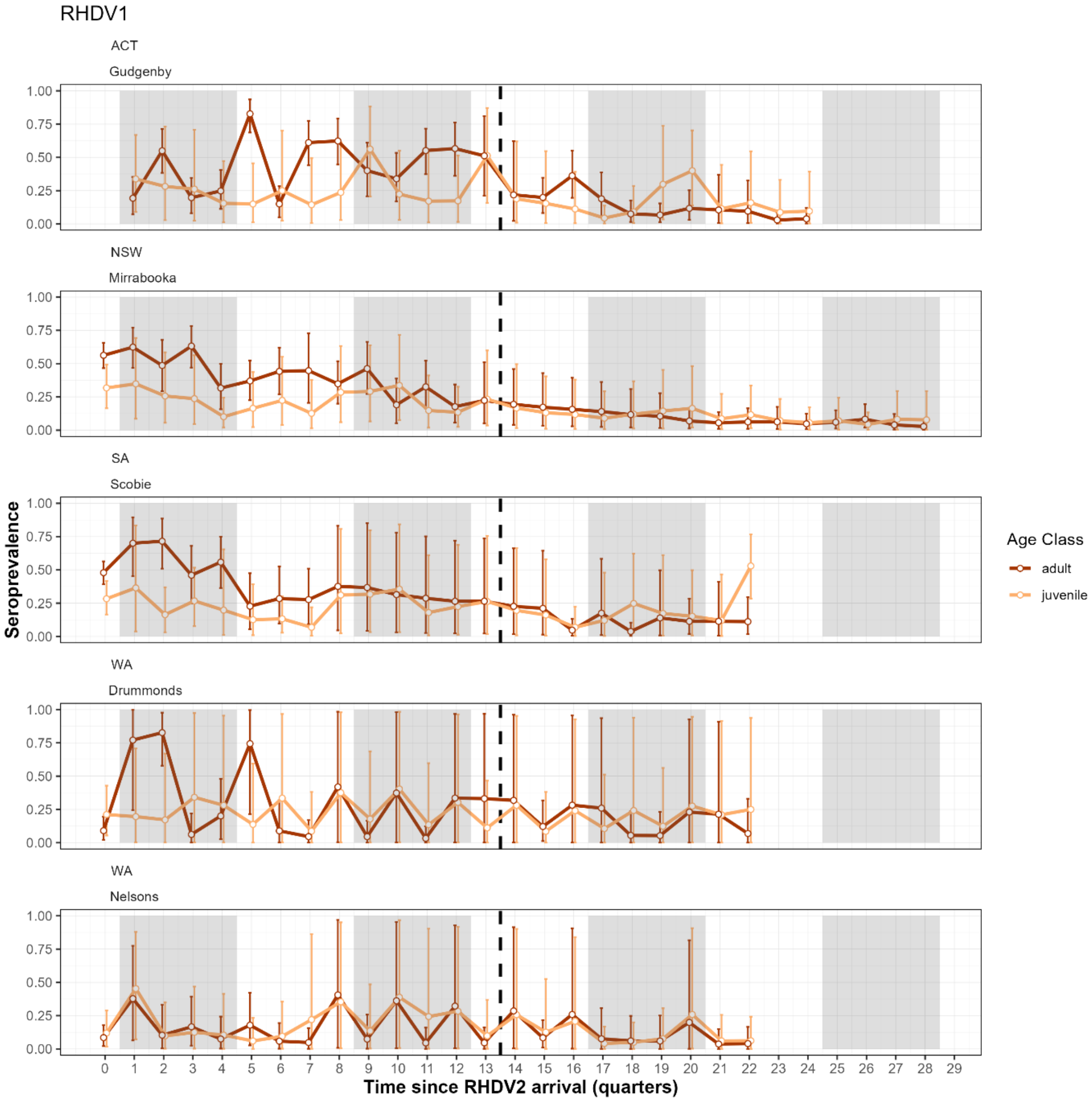

Figure S1. Estimated average trends in the seroprevalence of RHDV1 for juvenile (≤ 150 days old) and adult (> 150 days) rabbits following the arrival of RHDV2 at the five sites where monitoring continued post 2018. Dashed vertical black line shows the start of new data added since Ramsey et al (2020).

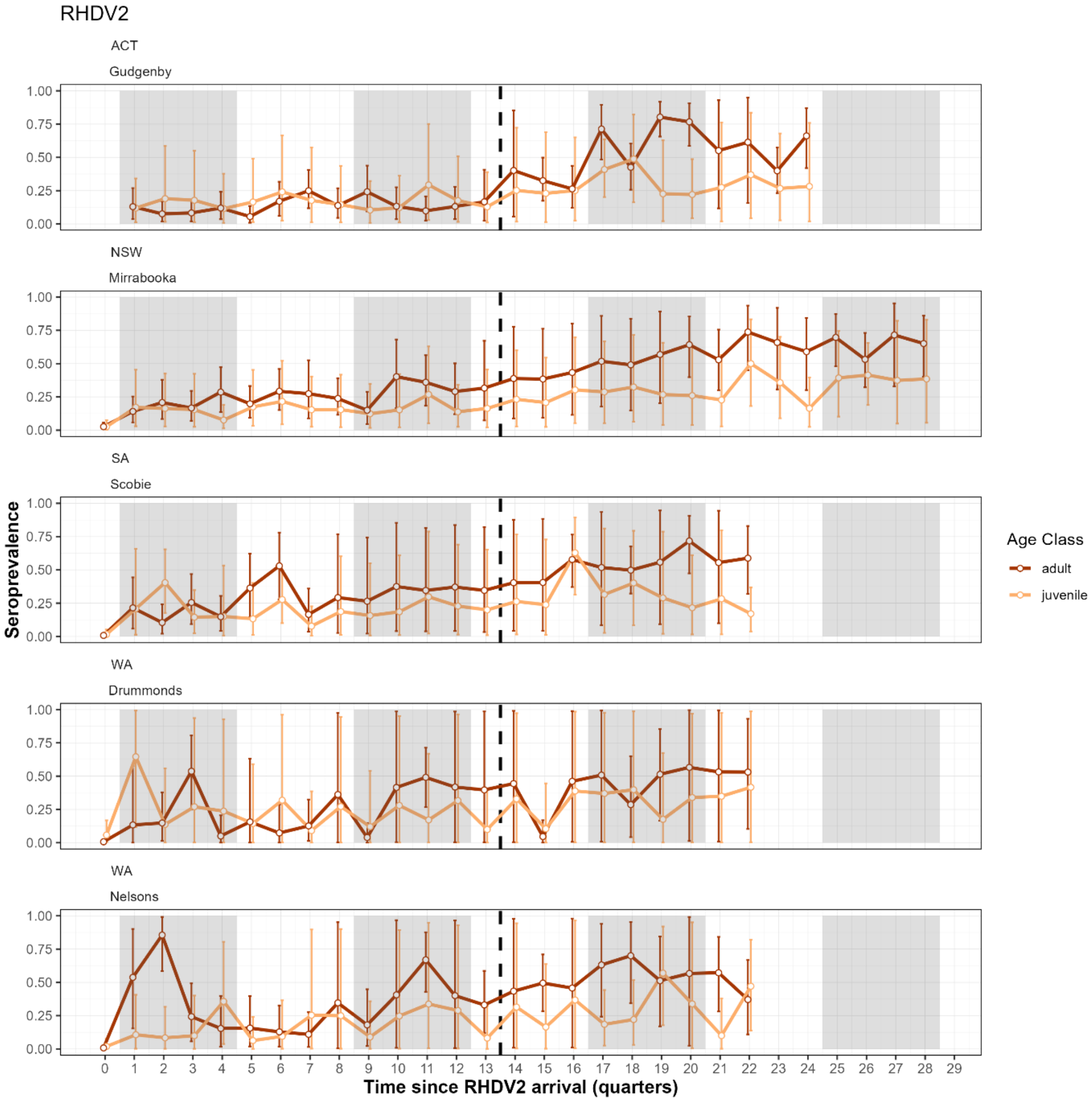

Figure S2. Estimated average trends in the seroprevalence of RHDV2 for juvenile (≤ 150 days old) and adult (> 150 days) rabbits following the arrival of RHDV2 at the five sites where monitoring continued post 2018. Dashed vertical black line shows the start of new data added since Ramsey et al (2020).

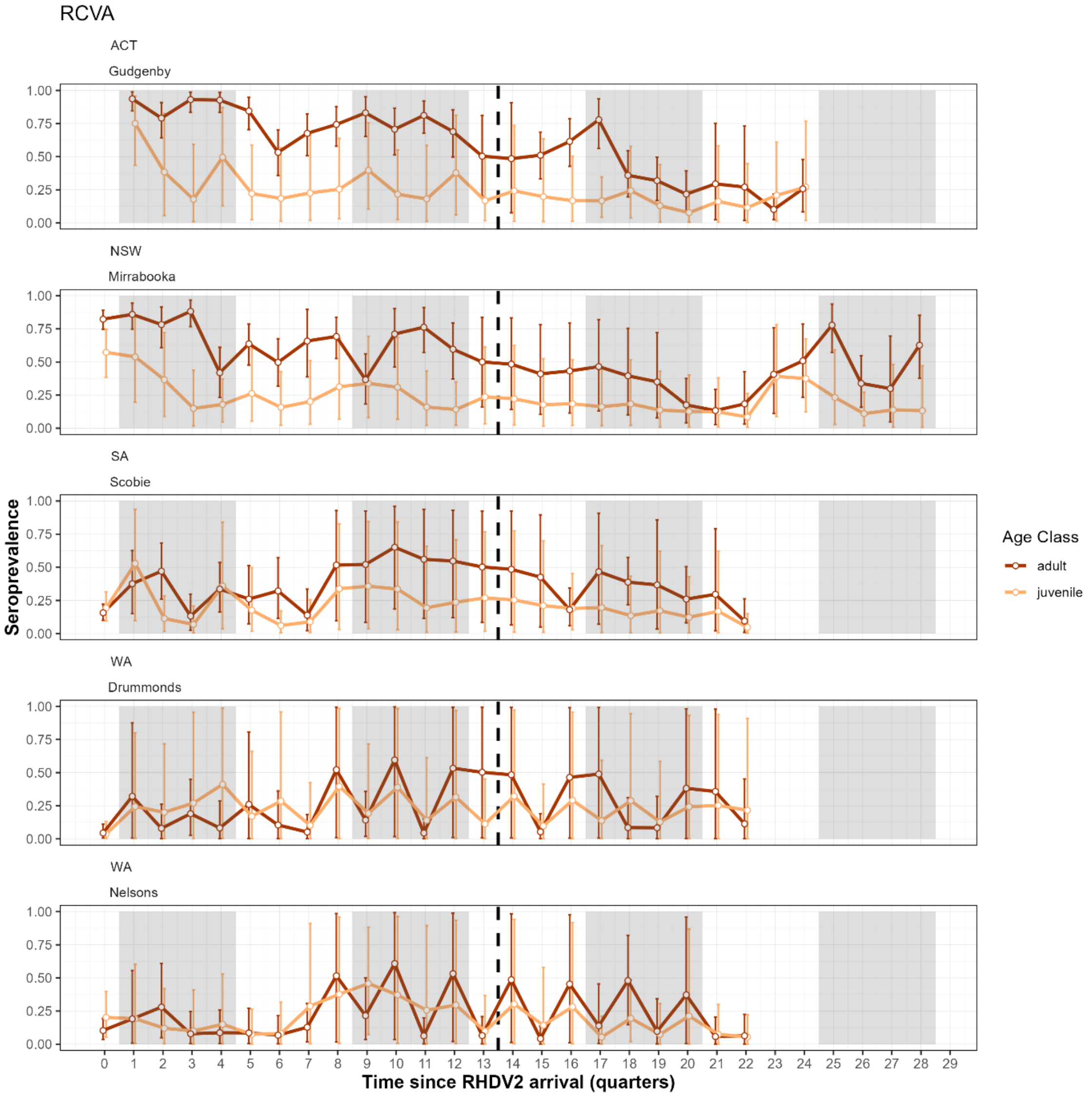

Figure S3. Estimated average trends in the seroprevalence of RCVA for juvenile (≤ 150 days old) and adult (> 150 days) rabbits following the arrival of RHDV2 at the five sites where monitoring continued post 2018. Dashed vertical black line shows the start of new data added since Ramsey et al (2020).
